# Loss of the virulence plasmid by *Shigella sonnei* promotes its interactions with CD207 and CD209 receptors

**DOI:** 10.1099/jmm.0.001297

**Published:** 2021-02-16

**Authors:** Bi-cong Wu, Njiri A. Olivia, John Mambwe Tembo, Ying-xia He, Ying-miao Zhang, Ying Xue, Cheng-lin Ye, Yin Lv, Wen-jin Li, Ling-Yu Jiang, Xi-xiang Huo, Zi-yong Sun, Zhong-ju Chen, Ji-chao Qin, An-yi Li, Chae Gyu Park, John D. Klena, Hong-hui Ding, Tie Chen

**Affiliations:** ^1^​ Department of Clinical Immunology, Tongji Hospital, Tongji Medical College, Huazhong University of Sciences and Technology, Wuhan, Hubei, PR China; ^2^​ Henan Provincial Center for Disease Control and Prevention, Zhengzhou, Henan, PR China; ^3^​ Department of Biological Sciences, Faculty of Science, Engineering and Technology, Chuka University, 109-60400, Kenya; ^4^​ Department of Paediatrics & Child Health, the University of Zambia – University College London Medical School at Zambia, Lusaka, Zambia; ^5^​ Clinical Research Center, Wuhan Pulmonary Hospital, Wuhan, Hubei, PR China; ^6^​ Department of Clinical Laboratory, The Central Hospital of Wuhan, Tongji Medical College, Huazhong University of Science and Technology, Wuhan, Hubei, PR China; ^7^​ Hubei Provincial Center for Disease Control and Prevention, Wuhan, Hubei, PR China; ^8^​ Department of Laboratory Medicine, Tongji Hospital, Tongji Medical College, Huazhong University of Sciences and Technology, Wuhan, Hubei, PR China; ^9^​ Key Laboratory of Hepatobiliary Surgery and Department of Hepatobiliary Surgery, Tongji Hospital, Tongji Medical College, Huazhong University of Sciences and Technology, Wuhan, Hubei, PR China; ^10^​ Laboratory of Immunology, Brain Korea 21 PLUS Project for Medical Science, Severance Biomedical Science Institute, Yonsei University College of Medicine, Seoul, Republic of Korea; ^11^​ Centers for Disease Control and Prevention, Atlanta, GE, USA

**Keywords:** antigen presenting cells (APCs), C-type lectins (CD207 and CD209), *Shigella sonnei*

## Abstract

**Introduction:**

*Shigella sonnei,* the cause of bacillary dysentery, belongs to Gram-negative enteropathogenic bacteria. *S. sonnei* contains a 210 kb virulence plasmid that encodes an O-antigen gene cluster of LPSs. However, this virulence plasmid is frequently lost during replication. It is well-documented that after losing the O-antigen and becoming rough strains, the Gram-negative bacteria may express an LPS core on its surface. Previous studies have suggested that by using the LPS core, Gram-negative bacteria can interact with several C-type lectin receptors that are expressed on antigen-presenting cells (APCs).

**Hypothesis/Gap Statement:**

*
S. sonnei* by losing the virulence plasmid may hijack APCs via the interactions of LPS-CD209/CD207.

**Aim:**

This study aimed to investigate if the *S. sonnei* rough strain, by losing the virulence plasmid, interacted with APCs that express C-type lectins of human CD207, human CD209a and mouse CD209b.

**Methodology:**

SDS-PAGE silver staining was used to examine the O-antigen expression of *S. sonnei* WT and its rough strain. Invasion assays and inhibition assays were used to examine the ability of *S. sonnei* WT and its rough strain to invade APCs and investigate whether CD209 and CD207 are receptors for phagocytosis of rough *S. sonnei *. Animal assays were used to observe the dissemination of *S. sonnei *.

**Results:**

*
S. sonnei* did not express O-antigens after losing the virulence plasmid. The *S. sonnei* rough strain invades with APCs, including human dendritic cells (DCs) and mouse macrophages. CD209 and CD207 are receptors for phagocytosis of rough *S. sonnei *. Expression of the O-antigen reduces the ability of the *S. sonnei* rough strain to be disseminated to mesenteric lymph nodes and spleens.

**Conclusion:**

This work demonstrated that *S. sonnei* rough strains – by losing the virulence plasmid – invaded APCs through interactions with CD209 and CD207 receptors.

## Introduction

*Shigella* spp.*,* the cause of bacillary dysentery, belong to Gram-negative invasive enteropathogenic bacteria that can penetrate mucosal surfaces of guts. The ingestion of as few as 100 bacteria is enough to cause bacillary dysentery [1]. Shigellosis represents a significant public health burden in developing countries, with about 160 million cases occurring annually, predominantly in children under the age of 5 years [2]. The *Shigella* genus comprises four species, including *Shigella dysenteriae, Shigella flexneri, Shigella boydii* and *Shigella sonnei *, and *S. flexneri* and *S. sonnei* cause the most infections [3].

The ratio of species dominance is highly dependent on the socio-economic conditions of an area. In developing countries, including those of sub-Saharan Africa and certain countries in Asia, *S. flexneri* is the dominant cause of shigellosis, responsible for over 60 % of infections [4]. In developed countries, such as Europe and North America, *S. sonnei* causes around 80 % of shigellosis cases [5]. Countries undergoing socio-economic improvements are also experiencing a change in infections from *S. flexneri* to *S. sonnei* [6]. It appears that the frequency of *S. sonnei* isolation directly correlates with per capita gross domestic product (GDP) [7]. The primary cause of this association is not fully understood. One hypothesis is that exposure to unsanitized drinking water systems in developing countries results in *Plesiomonas shigelloides* infection, and hence in natural immunity against *S. sonnei. P. shigelloides* serotype O17 has a lipopolysaccharide O-antigen identical to that of *S. sonnei* [8]. Also, *S. sonnei* has been shown to possess a functional type 6 secretion system (T6SS), which provides a niche-specific competitive advantage for *S. sonnei* over *S. flexneri* [6].

**Fig. 1.**
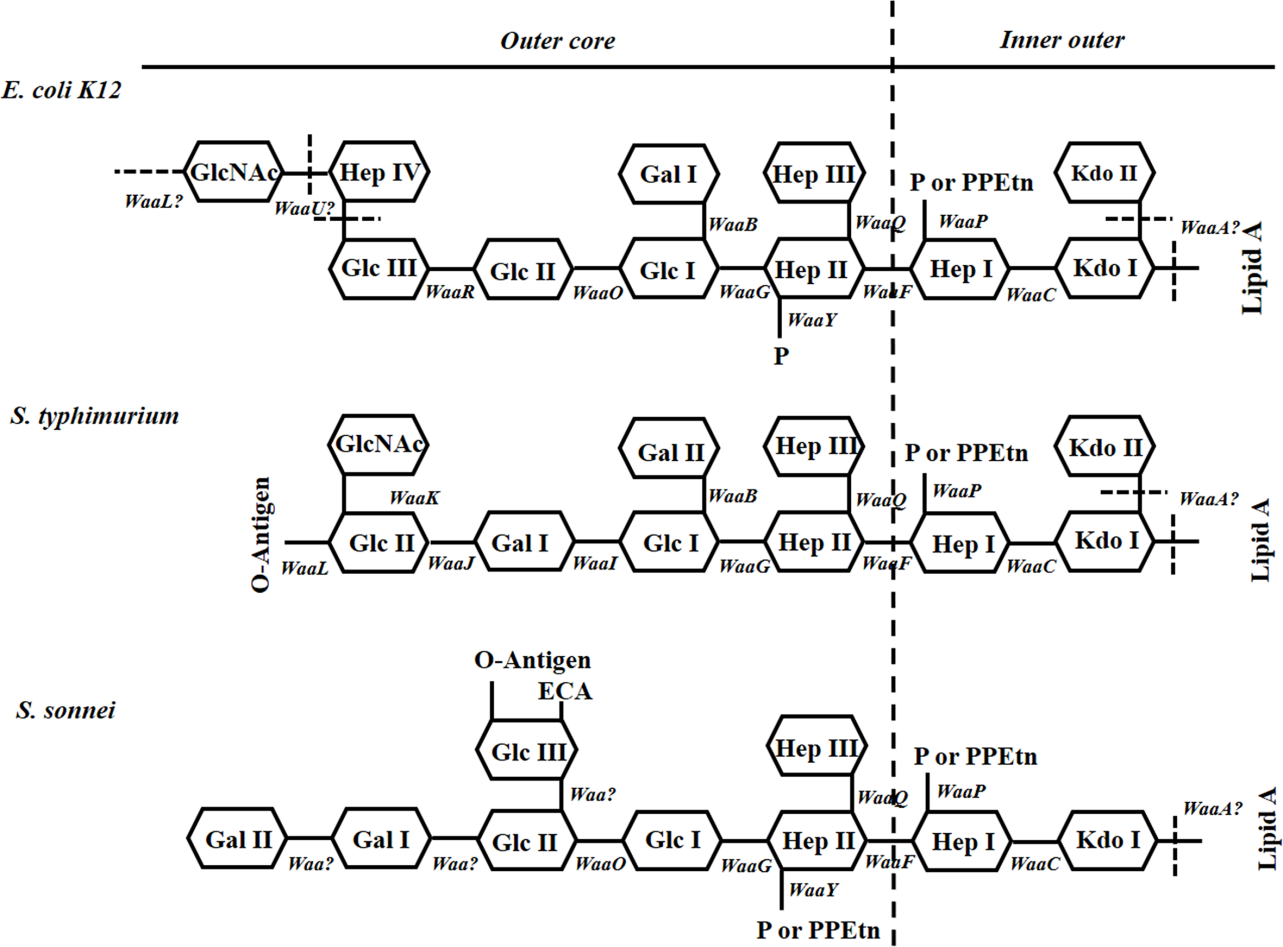
Structures of inner- and outer-core regions of the LPS or LOS of *E. coli* K12, *S. typhimurium* and *S. sonnei* and the genes involved in their synthesis. Genes involved in the biosynthesis of core LPS are shown at their approximate site of action (solid line). The sites, which are variably substituted or still under investigation, are indicated by dashed lines. The abbreviations in this figure are as follows: GlcNAc, *N*-Acetylglucosamine; Glc, glucose; Hep, heptose; Gal, galactose; P, phosphate; PPEtn, phosphoethanolamine; KDO, 2-keto-3-deoxyoctonate. It should be noted that *E. coli* K12 does not possess O-Ag.

Most Gram-negative enteropathogenic bacteria contain LPSs located in the outer membrane. The LPSs are composed of three covalently linked domains: lipid A, which is embedded in the outer membrane; the oligosaccharide core (Fig. 1); and the O-polysaccharide or O-antigen, which cover the bacterial surface [9]. The O-antigen is one of the essential components for bacterial survival during infection. For example, *Salmonella enterica, Francisella tularensis* and *Burkholderia cepacia* utilize the O-antigen to avoid phagocytosis and to resist lytic action of the complement system [10–12]. Unlike *S. flexneri *, the genes for O-antigen in *S. sonnei* are located on the large 210 kb virulence plasmid involved in invasion [13]. However, the large plasmid of *S. sonnei* is unstable and easily lost. Why does *S. sonnei* carry genes that control the synthesis of the somatic antigen and the expression of virulence on an unstable plasmid, and what are selective pressures to maintain the virulent state [14]? Maybe, *S. sonnei* has evolved to lose the virulence plasmid as an advantage during infection [15].

Rough Gram-negative bacteria bear shortened LPSs – referred to as lipooligosaccharides (LOS) – for which the oligosaccharide core is exposed to the extracellular environment [9]. Previous studies suggested that by using the LPS core, Gram-negative bacteria can interact with human Langerin (CD207) and human DC-SIGN (CD209), expressed on antigen-presenting cells (APCs) [16–22]. It is well-documented that human DC-SIGN is also a receptor for HIV GP-120 that hijacks DC-SIGN to be captured by APCs and trafficked to target cells such as CD4 lymphocytes [23–25].

*Shigella* initially crosses the epithelial layer of the colorectal mucosa via M cells. M cells are capable of transcytosing lumenal antigens into the subepithelial space where gut-associated lymphoid tissue and/or APCs, such as dendritic cells (DCs) and macrophages, are located [26]. The APCs of colonic and rectal mucosa can act as ‘Trojan horses’ allowing crossing of the intestinal epithelial barrier [27, 28].

In this study, we investigated if *S. sonnei* rough, rather than smooth, strains interacted with human DCs from guts, murine primary macrophages and the CHO cell lines that express C-type lectins of human CD207, human CD209a and mouse CD209b.

## Methods

### Bacterial strains

Bacterial strains used in this study are listed in Table 1. *E. coli K12* strain CS180 contains core oligosaccharides but lacks the O-antigen. CS1861 is the strain of CS180 harbouring pSS37, a plasmid containing all genes necessary for expression of the *Shigella dysenteriae* serotype 1 O-antigen [29]. *E. coli* strains were cultured on Luria–Bertani (LB) agar at 37 °C overnight. *Y. pseudotuberculosis* (Y1) is a serotype O:1a strain, lacking the virulence plasmid (pYV); the strain was obtained from the Centers for Disease Control (GA, USA), and used as a control strain for invasion since this bacterium invades almost all epithelial cell lines via an invasin-integrin interaction [30–32]. This strain has been used as a positive invasion control previously and was cultured on LB agar at 26 °C overnight [33]. *S. sonnei *, a clinical strain isolated from a patient with dysentery in Hubei Provincial Center for Disease Control and Prevention, Wuhan, PR China, was incubated in tryptic soy broth (TSB) at 37 °C overnight. *S. sonnei* rough strain without the 210 kb virulence plasmid was obtained from TS broth containing 0.6 % yeast extract with 1.5 % agar and 0.003 % Congo red [15, 34]. Rough O^+^ is a derivative of *S. sonnei* rough strains that harbour pSS37, a plasmid containing all of the genes necessary for expression of the *S. dysenteriae* serotype 1 O-antigen [29]. The three *S. sonnei* strains are isogenic. *S. sonnei* rough and rough O^+^ strains were cultured on LB agar with the corresponding antibiotic at 37 °C overnight.

**Table 1.** Bacterial strains and cell lines used in this study

Strains	Genotypes (phenotypes)	Refs
*E. coli* K 12		
CS180	WT (rough)	[29, 53, 54]
CS1861	CS180-O antigen (smooth)	
***Y. pseudotuberculosis***		
Y 1	O:1a, WT expressing invasin but with PYV plasmid naturally cured (smooth)	[30–32, 55]
*** S. sonnei ***		
*Ss-*WT	WT (smooth)	This study
*Ss-*rough	without 210 kb virulence plasmid (rough)	This study
Rough O^+^	*S. sonnei-*rough with pSS37 (smooth)	This study
**Cell lines**	**Characteristics**	
CHO-NEO cells	Control cell line, which expresses the neomycin resistance gene only	
CHO-mSIGNR1	Generated by transfecting CHO cells with mSIGNR1 cDNAs for stable surface expression	
CHO-hDC-SIGN	Generated by transfecting CHO cells with hDC-SIGN cDNAs for stable surface expression	
CHO-hLangerin	Generated by transfecting CHO cells with hLangerin cDNAs for stable surface expression	

### Cell lines

Cell lines used in this study are listed in Table 1. CHO-mSIGNR1, CHO-hDC-SIGN and CHO-hLangerin cell lines were generated by transfecting CHO cells with corresponding C-type lectin cDNAs. Transfection was followed by G418 (1.5 mg ml^− 1^) selection and screening for stable surface expression [35]. CHO-NEO cells were used as a control cell line that expresses the neomycin resistance gene only.

### LPS isolation and SDS-PAGE silver staining

The LPS extracts were isolated using the Lipopolysaccharide Extraction Kit (iNtRON Biotechnology, Korea), performed according to the manufacturer’s instructions. The *E. coli* strains CS180 and CS1861, which show rough LPS (without O-antigen) and smooth LPS (with O-antigen), respectively, were used as control strains. After purification, the LPS extracts were analysed by 12 % Bis-Tris SDS-PAGE and silver stained using the SilverQuest Silver Staining Kit (Invitrogen).

### Isolation of mouse peritoneal macrophages

The experiments for isolating mouse peritoneal macrophages have been described previously [20]. After the mice were euthanized, abdomens were immediately exposed, cleaned with 75 % ethanol and opened with scissors; 5 ml of RPMI was injected into the intraperitoneal cavity. The mouse abdomen was gently massaged for 2 min and then lavage fluid was collected. The suspension containing the macrophages was seeded onto six-well plates, in which each well contained a 1.5 cm diameter glass cover-slide, and placed in a CO_2_ incubator for 1.5 h. The cell layers were washed three times to remove non-adherent cells. Macrophages were then removed from the plastic surface by incubating with citrate saline and re-seeded for interaction assays.

### Isolation of human gut DCs

Human intestinal segments were obtained from patients who were undergoing gastrointestinal surgery and provided informed consent. Purification of gut DCs has been described previously [22, 36]. Samples were collected in ice-cold Dutch modification of RPMI 1640 supplemented with 10 % FBS, 2 mM l-glutamine, gentamicin (25 µg ml^−1^), and penicillin/streptomycin (100 U ml^−1^). The samples were incubated for 20 min at room temperature in calcium and magnesium-free HBSS containing 1 mM DTT. To remove the epithelium, biopsies were transferred to HBSS containing 1 mM EDTA and incubated for 30 min on a shaker at 37 °C. To continue the isolation of gut DCs, the tissue was digested with 1 mg ml^−1^ collagenase D in HEPES-buffered RPMI 1640 containing 20 µg ml^−1^ DNase I and 2 % FCS at 37 °C on a shaker for 90–180 min. Mononuclear cells were separated (650 g, 20 min, room temperature) on Ficoll-Paque and washed in complete medium. The isolated cells were labelled with anti-CD11c^+^ (Biolegend) and anti-hDC-SIGN antibodies (Pharmingen) and then examined by flow cytometry.

### Invasion assays

The invasion assays have been described previously [37]. Briefly, host cells (CHO, hLangerin, mSIGNR1 and hDC-SIGN) were plated in a cell culture flask. The degree of cell fusion was 80 %. The cells were suspended in RPMI medium supplemented with 2 % foetal calf serum at a concentration of 1×10^5^ ml^−1^ and were added to 24-well plates; 12–24 h later, cells were washed three times. The 500 µl RPMI medium supplemented with 2 % foetal calf serum was added to 24-well plates. After the addition of 50 µl of bacterial suspensions at a concentration of 5×10^6^ c.f.u. ml^− 1^, cells were incubated for 2 h at 37 °C in the presence of 5 % CO_2_.

To determine the internalization of bacteria, gentamicin – which kills extracellular bacteria but cannot penetrate host cells – was added to each well at a final concentration of 100 µg ml^− 1^ and the cultures were incubated for 60 min. The cells were washed three times to remove the gentamicin. Cells were suspended in phosphate-buffered saline containing 0.25 % Triton, after which the cells were diluted and plated on LB plates with corresponding antibiotics. The level of internalization of bacteria in the host cells was calculated by determining the c.f.u. recovered from lysed cells. All experiments were performed in triplicate, and the data are expressed as means±standard errors.

For the inhibition assay, the concentration of cells was 2×10^5^ ml^−1^. Anti-mSIGNR1 (5 µg ml^− 1^) antibody, anti-hLangerin (5 µg ml^− 1^) antibody (Pharmingen), and mannan (500 µg ml^− 1^) (Sigma-Aldrich) were added 20 min before the addition of bacteria. The concentrations used were determined based on our preliminary data and were selected based on the fact that, at these concentrations, the compounds exerted no effects on the survival of bacteria or host cells, as previously shown [17, 18].

### Animal assays

All animal experiments were carried out in strict accordance with the Institutional Animal Care and Use Committees (IACUCs) and Institutional Review Board (IRB) of Tongji Hospital, Tongji Medical College, PR China. C57BL/6 female mice at the age of 5–6 weeks were purchased from Wuhan University Animal Center. Mice were housed in animal facilities at Tongji hospital in direct accordance with guidelines drafted by the Animal Care Committees of Tongji Hospital. For bacterial infection, three groups (six mice/group) of C57BL/6 mice were infected with bacteria (1×10^6^ c.f.u.) treated with corresponding antibiotics through an intraperitoneal route to observe dissemination.

To confirm bacterial colonization in infected tissues, spleens and MLNs were collected after 24 h from intraperitoneally challenged mice. Tissues were removed and vigorously washed in PBS with gentamicin (100 μg ml^−1^) to remove bacteria that were simply attached but had not invaded the tissues [38]. Tissues were then mechanically homogenized in 1 ml of 1 % Triton, diluted, and plated onto plates containing antibiotics. Colonies were counted after 18 h of culture at 37 °C.

### Statistical analyses

All statistical analyses were completed using Prism software, version 6 (Graph Pad, San Diego, CA, USA). Statistical significance was assessed using Student’s *t*-test for the univariate analysis of two sets of data and two-way ANOVA for multiple comparisons. *P* <0.05 was considered statistically significant.

## Results

### *S. sonnei* without the virulence plasmid did not express the O-antigen

**Fig. 2.**
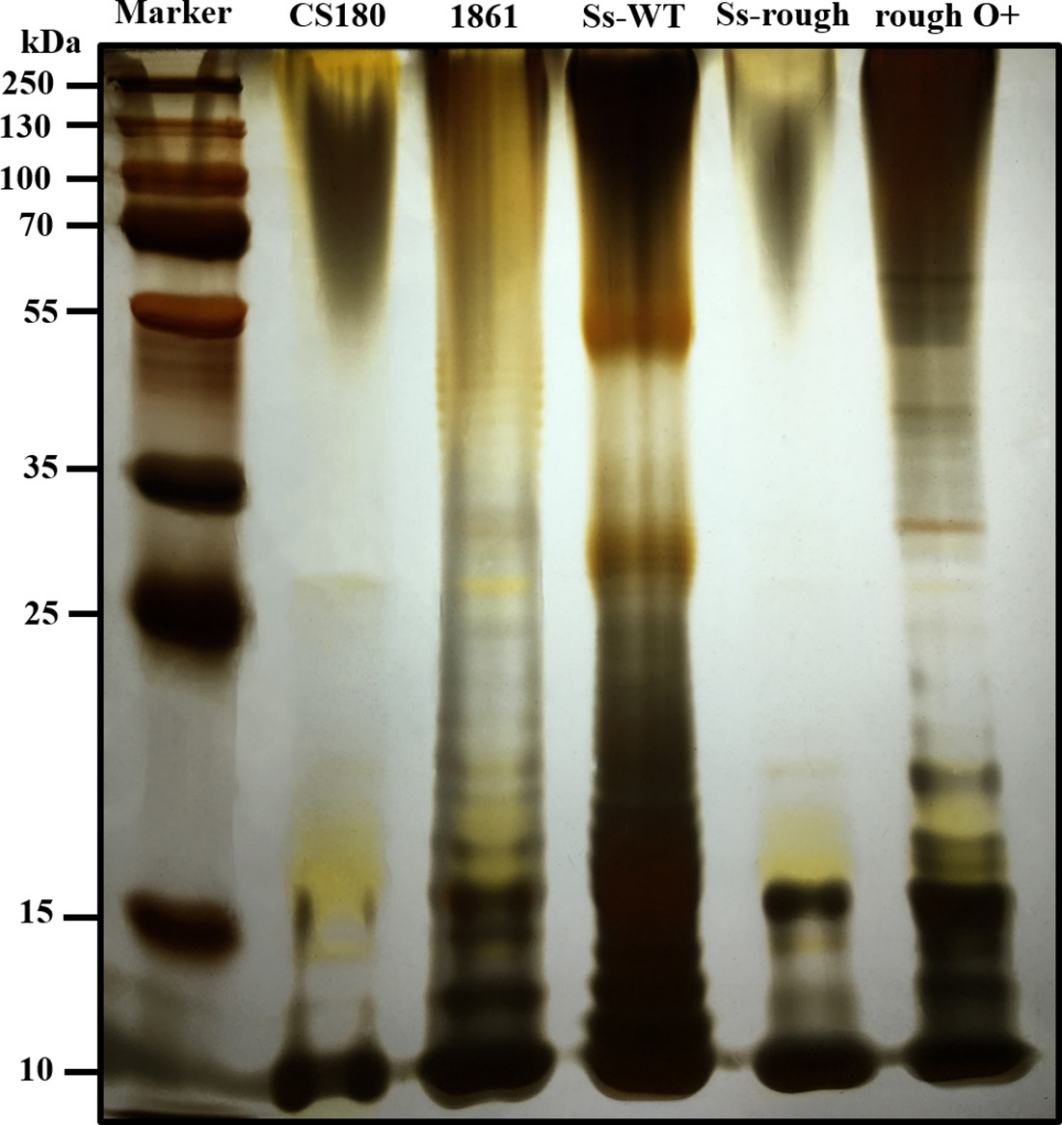
Examination of O-antigen expression of *S. sonnei* WT and its rough strain. Silver staining of the LPS of *S. sonnei* strains. Controls: *E. coli* strains CS180 and CS1861, which show rough LPS (without O-antigen) and smooth LPS (with O-antigen), respectively. *S. sonnei* strains: Ss-WT has the virulence plasmid to express the O-antigen. Ss-rough and rough O+, which show the rough LPS (without O-antigen) and smooth LPS (with O-antigen), respectively.

SDS-PAGE silver staining was used to examine the O-antigen expression of *S. sonnei* WT and its rough strain. The *E. coli* strains K-12 CS180 (lacking the O-antigen) and CS1861 (CS180 expressing an O-antigen) were used as controls [29]. As shown in Fig. 2, the LPS extract of *S. sonnei* WT had a typical LPS ladder with a predominant chain length of 20 to 25 O-Ag repeating units; *S. sonnei* rough strain (lacking the O-antigen-encoding virulence plasmid) had only the low molecular weight band corresponding to the LPS core-lipid A moieties. For rough O+, which harboured the pSS37 derivative of CS1861, the O-Ag repeating units were identical to CS1861.

### Rough *S. sonnei* invades human DCs and mouse macrophages

**Fig. 3.**
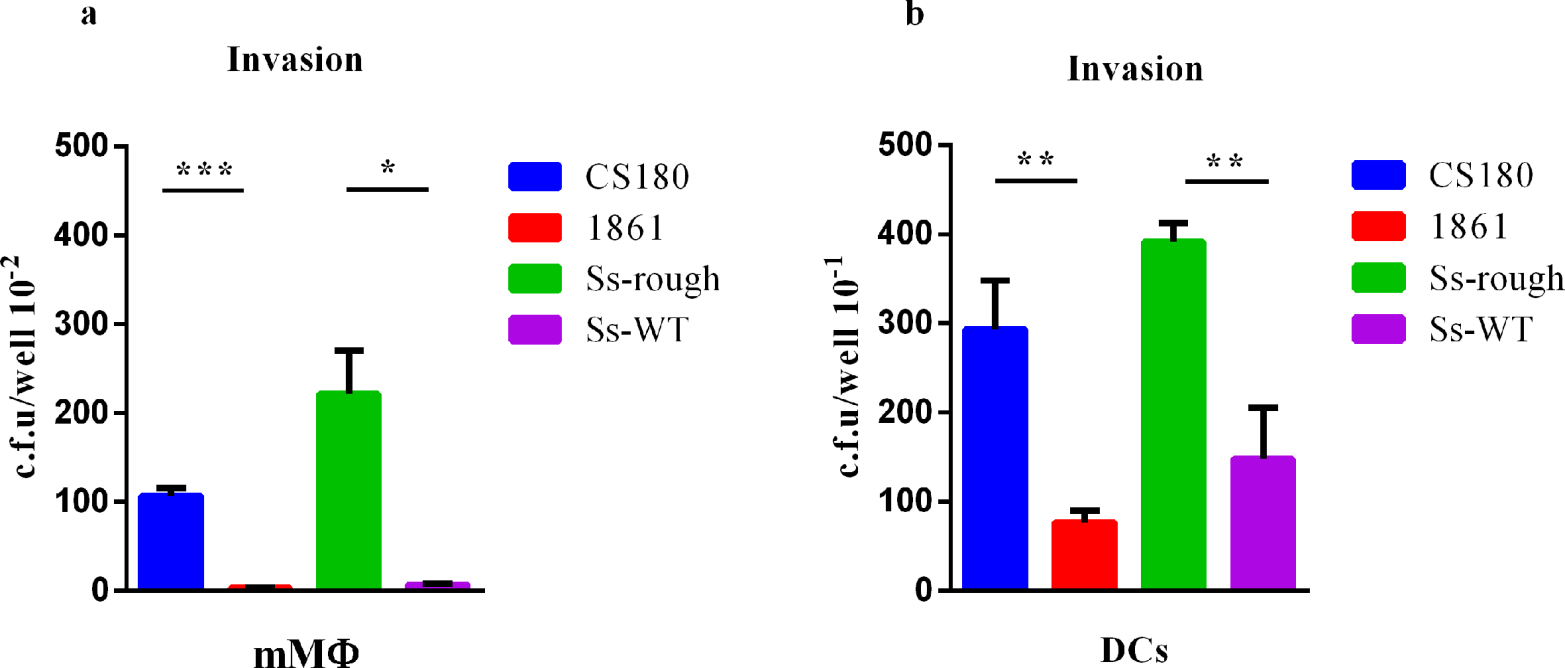
Human gut dendritic cells (DCs) and mouse macrophages (mMΦ) phagocytose the rough strains *S. sonnei *. Gentamicin protection assays were used to determine the invasion rates of two sets of the Gram-negative bacteria *S. sonnei* (Ss-WT, Ss-rough and rough O+) and *E. coli* K-12 (CS180 and CS1861) into mMΦ (a) or DCs (b). The number of phagocytosed bacteria was determined by counting c.f.u. recovered following gentamycin treatment. *P <0.05; **P <0.01; ***P <0.001.

We examined the ability of *S. sonnei* WT and its rough strain to invade DCs. The *E. coli* strains K-12 CS180 (an avirulent strain with the core LPS exposed) and CS1861 (CS180 expressing an O-antigen) were used as controls [17, 39]. Results from the gentamicin protection assays (Fig. 3a, b) showed that all of these *S. sonnei* strains were phagocytosed by DCs to some extent, and *S. sonnei* rough strains were taken up more than WT. Previously, it has been reported that the central hydrophobic portion of IpaC on virulence plasmids, the membrane-spanning domain, was critical for entry of *Shigella* into macrophages [40], suggesting that *S. sonnei* rough strains use different invasive mechanisms to hijack DCs.

### CD209 and CD207 are receptors for phagocytosis of rough *S. sonnei*

**Fig. 4.**
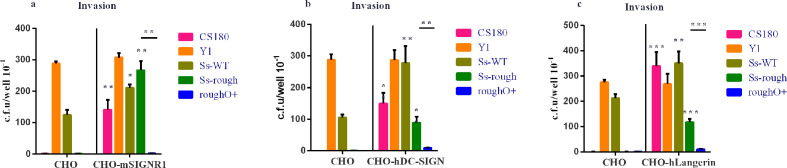
Phagocytosis of *S. sonnei* strains with transfecting CHO cells. The phagocytosis of two sets of bacteria *E. coli* K-12 (CS180 and CS1861) and *S. sonnei* (Ss-WT, Ss-rough and rough O+) by CHO-mSIGNR1 (a), CHO-hDC-SIGN (b) and CHO-hLangerin (c) cells were analysed. Bacteria and CHO transfectants were incubated together for 2 h, and extracellular bacteria were killed with 100 µg ml^− 1^ (final concentration) gentamicin. CHO-NEO cells were used as the negative control cell line. The number of phagocytosed bacteria was determined by counting c.f.u. recovered following gentamicin treatment. *P <0.05; **P <0.01; ***P <0.001.

To further study the interaction of DCs with *S. sonnei* rough strains, stably transfected mSIGNR1/hDC-SIGN/hLangerin CHO cell lines were used to test their ability to bind and internalize *S. sonnei* rough strains. *Y. pseudotuberculosis* (Y1) was used as a control since it invaded almost all epithelial cell lines via an invasion-integrin interaction [18, 31, 33]. Results showed that both CS180 and *S. sonnei* rough strains effectively invaded CHO-mSIGNR1 (Fig. 4a), CHO-hDC-SIGN (Fig. 4b) and CHO-hLangerin (Fig. 4c), but not the CHO. This demonstrated that *S. sonnei* rough strains interacted with mSIGNR1, hDC-SIGN and hLangerin, as the *S. sonnei* rough-mediated invasion was also blocked by the expression of O-antigen.

### Inhibition of receptor-mediated phagocytosis of *S. sonnei* rough strains by an anti-mSIGNR1/anti-hLangerin antibody

**Fig. 5.**
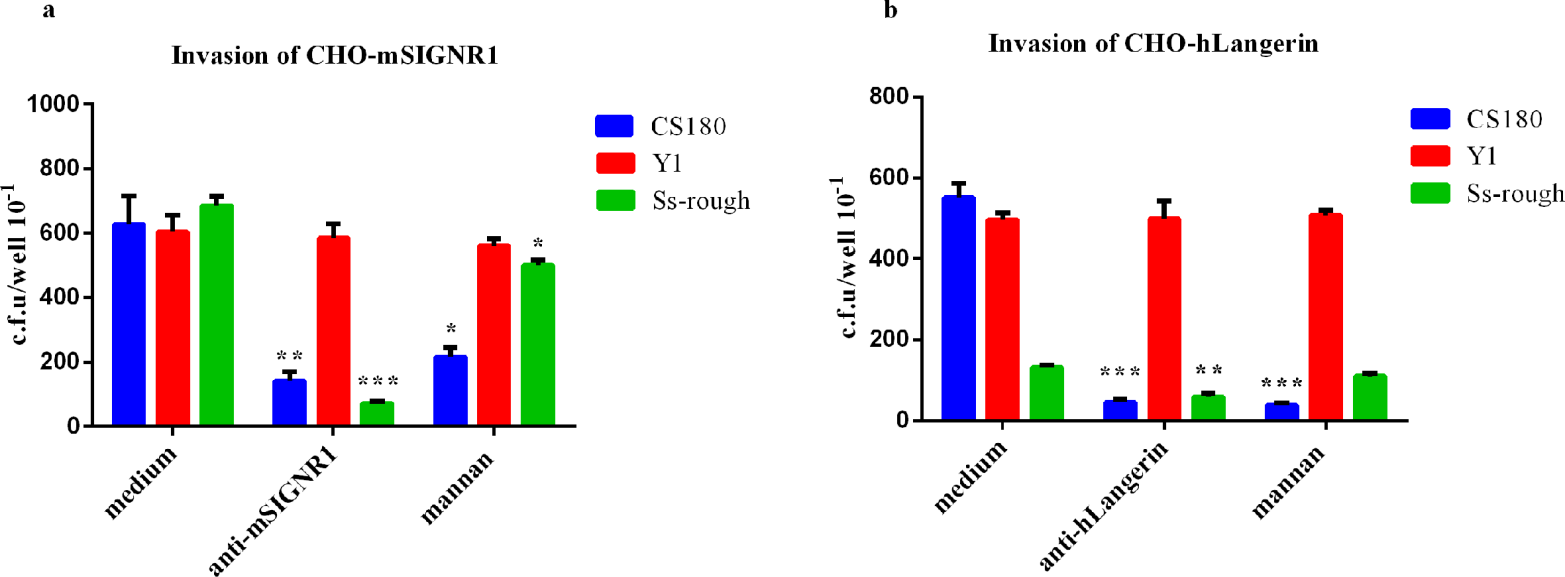
Inhibition of receptor-mediated phagocytosis of *S. sonnei* rough strain by antibodies. *S. sonnei* rough strain cultured at 37 °C was incubated with CHO-mSIGNR1 (a) and CHO-hLangerin (b) for 2 h in the presence or absence of anti-mSIGNR1, anti-hLangerin antibodies and mannan. All reagents were added to the media 20 min before the addition of bacteria. The phagocytosis rate of Ss-rough was determined by the recovery of bacteria following gentamicin treatment. *Y. pseudotuberculosis* serotype O:1a was used as a control strain that shows core-independent invasion of CHO cells. *P <0.05; **P <0.01; ***P <0.001.

To verify specific interactions of *S. sonnei* rough and hCD207/CD209 receptors, anti-mSIGNR1/anti-hLangerin antibodies and mannan (an antagonist of mannose receptors) were used. As shown in Fig. 5, when antibodies were applied, the phagocytosis of *S. sonnei* rough strains by transfected CHO cell lines was significantly reduced. This suggests that the mSIGNR1 and hLangerin have a role in the interaction between DCs and *S. sonnei* rough strains. Mannan still inhibited the interactions of CS180 with CD209 and hCD207 (Fig. 5a), as shown in our previous publications [18–20]. However, mannan did reduce the interaction of *S. sonnei* rough strains and CD209, but not the hCD207 receptor (Fig. 5b), suggesting that in addition to the LPS core other sugar residues exposed on the surfaces of *S. sonnei* rough strains may also mediate their interactions.

### Expression of O-antigen reduces the ability of *S. sonnei* rough strain to be disseminated to mesenteric lymph nodes and spleens

**Fig. 6.**
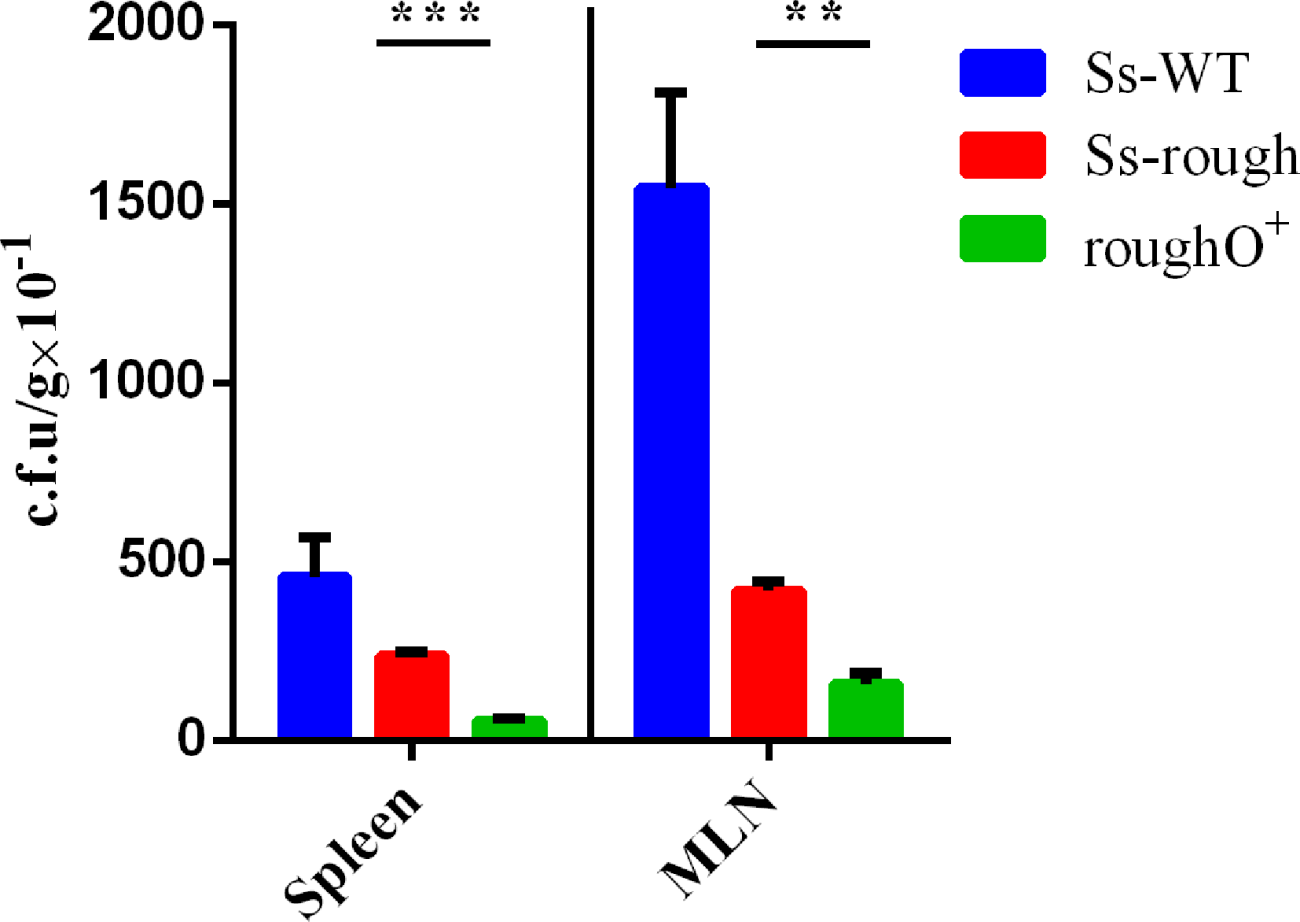
*
S. sonnei* strains disseminate in C57BL/6 female mice after intraperitoneal injection. Ss-WT, Ss-rough and rough O+ strains were inoculated in mice following the procedures described in Methods. After 24 h, the mice were killed, and the spleens and MLNs were separated, homogenized and spread on corresponding plates. The dissemination rate represents the c.f.u. recovered from spleens and MLNs. **P <0.01; ***P <0.001.

To determine whether *S. sonnei* can be disseminated to local lymph nodes and spleens, mice were infected via the intraperitoneal route. Fig. 6 shows that all *S. sonnei* WT, rough and rough O^+^ strains were isolated in spleens and MLNs, but *S. sonnei* rough strains were recovered from spleens and MLNs in higher numbers than rough O^+^ strains. indicating that the expression of O-antigen reduced the ability of *S. sonnei* to be disseminated. Most likely, because of the invasive genes on virulence plasmids, *S. sonnei* WT had a strong ability to be disseminated.

## Discussion

C-type lectins have been studied for their interactions with bacteria, viruses and parasites [41–43]. Both DC-SIGN and Langerin recognize carbohydrate structures with high mannose specificities, therefore, both bind HIV-1 gp120 [44]. Langerin induces the formation of the Birbeck granules in Langerin^+^ cells, and mediates HIV-1 degradation, whereas DC-SIGN mediates HIV-1 transmission [45, 46]. DC-SIGN can bind and readily internalize rough *E. coli*, *Klebsiella pneumonia*, *Mycobacterium tuberculosis *, *S. typhimurium *, *Y. pestis* and *Y. pseudotuberculosis* [17, 18, 20, 39, 47–49]. This CD209-LPS core leads to bacterial dissemination and persistent infection in general [19–22]. We, however, found that DC-SIGN/Langerin mediated the phagocytosis of *S. sonnei* rough strains, which was not able to cause disease after losing the virulence plasmid [14].

This virulence plasmid is essential for *S. sonnei* pathogenicity [50]. Specifically, infection and dissemination are tightly orchestrated by the IpaB and VirG proteins encoded on the virulence plasmid [51]. After losing the virulence plasmid, *S. sonnei* rough strains fail to escape from macrophages [40], and the CD209/hCD207-* S. sonnei* rough interactions most likely result in the clearance of rough *S. sonnei* during infection. As shown in Fig. 6, immune organs, such as spleens and MLNs, displayed higher levels of *S. sonnei* rough internalization compared to rough O^+^. Functioning as the shield for the O-antigen, it appears that recovery of rough O^+^ bacteria from spleens and MLNs is seen as an indication of partial virulence.

However, a recent paper indicated that deletion of toxin-antitoxin systems in the evolution of *S. sonnei* as a host-adapted pathogen reduces the metabolic burden for *S. sonnei* growth, reflecting the ongoing transition of *S. sonnei* into an obligate pathogen that is less dependent on survival outside a mammalian host than other species of *Shigella* [15]. Moreover, intracellular *Shigella* can remodel its LPS, dampen the innate immune recognition, and evade inflammasome activation [1]. Recent studies by Lugo-Villarino *et al*. demonstrated that DC-SIGN has an anti-inflammatory role in macrophages in response to pathogens [52]. Therefore, based on the CD209/hCD207*–S. sonnei* rough interactions – most likely result in the clearance of rough *S. sonnei* – we hypothesize that *S. sonnei* rough strains may provide for less immune system-based killing for losing the plasmid during infection (which contributes to *S. sonnei* WT pathogenicity). Future studies may focus on verifyfing the mechanisms of immune system-based killing for losing the plasmid and helping the survival of WT *S. sonnei *.
